# Early outcome of Coronary Artery Bypass Graft Surgery in patients with significant Left Main Stem stenosis at a tertiary cardiac care center

**DOI:** 10.12669/pjms.314.7597

**Published:** 2015

**Authors:** Muhammad Sher-i-Murtaza, Mirza Ahmad Raza Baig, Hafiz Muhammad Azam Raheel

**Affiliations:** 1 Dr. Muhammad Sher-i-Murtaza, FCPS Surgery, FCPS CS. Cardiac Surgery Department, Ch. Pervaiz Elahi Institute of Cardiology, Multan – Pakistan; 2 Mr. Mirza Ahmad Raza Baig, B.Sc Hons. Cardiac Surgery Department, Ch. Pervaiz Elahi Institute of Cardiology, Multan – Pakistan; 3 Dr. Hafiz Muhammad Azam Raheel, Diploma in Anesthesia. Cardiac Surgery Department, Ch. Pervaiz Elahi Institute of Cardiology, Multan – Pakistan

**Keywords:** Coronary Artery Bypass Grafting, Left Main Stem disease

## Abstract

**Objective:**

Primary objective of this study was to evaluate the impact of significant left main stem (LMS) stenosis on the early outcome of coronary artery bypass graft (CABG) surgery.

**Methods:**

A Retrospective non-randomized analytical study was conducted in Cardiac surgery department, Chaudhary Pervaiz Elahi Institute of Cardiology (CPEIC) Multan, Pakistan. The data of patients who underwent isolated CABG at our institution from February 2008 to March 2014 were analyzed. Two thousand six hundred two (2602) patients of isolated CABG were divided into 2 groups according to the LMS disease. Group I (n=2088): without significant LMS disease and Group II (n=514): with LMS disease. Data was analyzed using SPSS V16. The groups were compared using Student’s t-test for numeric variables. Chi-square test and Fishers Exact test were used for categorical variables. P-value ≤ 0.05 was considered as significant difference.

**Results:**

Out of two thousand six hundred two, 2088 patients were in Non.LMS group (Control Group) and five hundred fourteen were in LMS Group (Study Group). Patients with LMS disease were older. In both groups there was no statistically significant difference regarding gender distribution, risk factors of IHD, pre-operative renal function and preoperative CKMB levels. Significant number 50 (9.7%) of patients were unstable in LMS group and they needed urgent surgery (p-value <0.0001). Need and duration for inotropic support and intra-aortic balloon counter-pulsation support were significantly high in LMS group (p-value <0.0001, 0.002, 0.003 respectively). Similarly Mechanical ventilation time and hospital stay were higher in LMS group. Incidence of pulmonary complications and operative mortality were significantly higher in LMS group (p-value 0.005 and 0.001 respectively). Mortality of CABG patients with significant left main coronary stenosis was 13 out of five hundred fourteen (2.5%) as compared to just 17 out of two thousand eighty eight (0.8%) in control group.

**Conclusion:**

This study showed that significant LMS disease is an independent risk factor for early cardiopulmonary morbidity and mortality after CABG surgery.

## INTRODUCTION

A significant Left Main Stem (LMS) stenosis is considered when there is reduction of ≥ 50% of the vessel diameter at coronary angiogram. Significant LM stenosis occurs in about 6% of patients undergoing coronary angiography, 1 and in 30% of coronary artery bypass grafting (CABG) patients.^2^ Isolated Left main stem stenosis occurs in only 6% to 9% of patients, out of these over 70% to 80% of patients also have associated multivessel Coronary Artery Disease.^1^,^3^ Patients with significant LMS disease are considered by many at high risk of mortality after CABG.^2^,^4^,^5^ However, in many mortality risk estimation scoring systems like Parsonnet, Additive and logistic Euro-system, no one have identified LMS disease as an independent risk factor for mortality.^6^ The significant LMS disease is a poor prognostic factor with a 3-year survival as low as 37% depending on the degree of stenosis, left ventricular function, and associated distal coronary artery disease.^7^ The magnitude of surgical benefit is influenced by both the degree of LMS stenosis and left ventricular function. The operative mortality is also associated with these factors as well as the emergent need for surgery, gender and left coronary dominance.^8^

CABG in presence of significantly diseased left main coronary artery is likely to pose many challenges and patients with significant LMS disease are likely to become hemodynamically unstable at time of induction and during cardiac manipulation at time of surgery. In this study, main focus was to see the impact of significant LMS disease on early outcome of CABG surgery.

## METHODS

A retrospective non-randomized analytical study was conducted in Cardiac surgery department, Chaudhary Pervaiz Elahi institute of cardiology (CPEIC), Multan, Pakistan. The CPEIC is a tertiary cardiac care center and is presently performing over 600 coronary artery bypass surgery annually. The study was conducted in strict compliance of the rules established by the revised Helsinki convention and had approval from the ethical committee of the institute. The data of patients operated from February, 2008 to March, 2014 were analyzed. The data was retrieved from Cardiac Surgery DATA BASE of the institution. More than 3000 patients’ characteristics were prospectively entered in our electronic database (CASCADE DATABASES, Lahore, Pakistan).

Patients undergoing isolated CABG were included in the study and they were divided in two groups according to significant LMS disease. Group I Patients without significant LMS disease (Control Group) and Group II: Patients with significant LMS disease (Study Group).

General anaesthesia was used in all patients. Patients were pre-medicated with oral dose of 3mg bromazepam the night before surgery. Anaesthesia was induced with intra-venous morphine (0.1mg/kg), midazolam (0.05-0.1 mg /kg), and propofol (1.0-2.5 mg/kg titrated according to the response. They were given atracuronium (1mg/kg) before endotracheal intubation. The anesthesia was maintained with sevoflorane/isoflurane. Both on-pump and off-pump coronary artery bypass surgery was done depending on stability of patient besides surgeon preference and experience.

Cold blood cardioplegia was used for myocardial protection in patients undergoing conventional CABG in both groups. The necessity of inotropic support and the choice of inotropic drugs to be administered during weaning from cardiopulmonary bypass (CPB) and in Intensive care unit (ICU) were noted. Inotropic support was defined as mild when dobutamine was administrated at a rate <5 ug/kg/min, moderate when dobutamine was administrated at 5-10 ug/kg/min and high dose when dobutamine was administrated at >10 ug/kg/min. Need of adrenaline or noradrenaline as inotropic support <0.06 ug/kg/min was considered as mild support, 0.06 to 1.0 ug/kg/min as moderate and >1 ug/kg/min was considered as high inotropic support.

Pre-op and maximum post-op CK-MB levels were noted. Enzymatic criteria was used to rule out peri-operative MI, a rise in CKMB Levels five times the designated reference value i.e. >125 IU/liter was considered as MI. The rise in serum creatinine levels two folds of preoperative value or the need for renal replacement therapy like hemo-dialysis was considered as renal complication. The development of significant pleural effusion and pneumothorax which need surgical intervention either paracentesis or chest tube insertion, Adult Respiratory Distress Syndrome (ARDS) and Pulmonary Embolism was recorded as pulmonary complication. The immediate need of surgery prior to next available routine operative list was defined as emergency surgery. If surgery has to be performed on immediate available list or patient has to be kept admitted in hospital to perform surgery was defined as urgent surgery. And patients whom routine operative time was given on outpatient basis was included in elective surgery.

The statistical analysis was carried out using SPSS (SPSS version 16, SPSS Inc, Chicago, IL). The preoperative, operative and postoperative characteristics were summarized using means and standard deviation for the numeric variables. The groups were compared using Student’s t-test for numeric variables. Chi-square test and Fishers Exact test were used to analyze categorical variables. The significance of differences between the groups was expressed as p-value and a value of ≤ 0.05 was considered significant.

## RESULTS

Preoperative, operative and postoperative characteristics of patients are summarized in Table-I & II. A total of two thousand six hundred two (2602) patients underwent isolated CABG at our institution and their characteristics were retrieved from electronic data base. There were 2088 patients in Non-LMS Group (Control Group) and 514 in LMS Group (Study Group). Patients with left main stem disease were older (57.74±9.71 years) as compared to patients without left main stem disease (55.33±9.59 years) (p-value <0.0001), majority of patients in both groups were in CCS (Canadian Cardiovascular Society) angina class III. However, significant number of patients were with unstable angina in LMS disease group (p-value <0.0001). Majority of patients (9.7%) underwent urgent surgery in LMS group (p-value <0.0001). In both groups, there was no statistically significant difference regarding gender distribution, risk factors of IHD, Body Mass Index, pre-operative renal function and preoperative CKMB levels. The extent of coronary artery disease and LV dysfunction were more severe in Non-LMS group (p-value <0.0001 and 0.001 respectively). Regarding preoperative operative mortality risk stratification scoring systems no difference was seen in both groups. More patients under went off pump CABG in non LMS group. Both groups showed no significant difference in aortic cross clamp time and total Bypass time.

**Table-I:** Comparison of demographic, echocardiographic and angiographic characteristics.

Name of Variable		Non-LMS group (Control Group)	LMS Group (Study Group)	P-Value
***Demographic Details***				
No. of Patients		2088	514	
Age (years)		55.33±9.59	57.74±9.71	<0.0001
Gender (%)	Male	1771 (84.8)	443 (86.2)	0.435
	Female	317 (15.2)	71 (13.8)
Body Mass Index		26.59±4.58	26.04±4.71	0.017
***Risk Factors of IHD***				
Diabetic history (%)		757 (36.3)	164 (31.9)	0.06
Smoking history (%)		809 (38.7)	184 (35.8)	0.22
History of Hypertension (%)		47.4	47.0	0.93
Family History (%)		458 (21.9)	112 (22.2)	0.90
History of hyper-cholestrolemia (%)		133 (6.4)	46 (8.9)	0.04
***Co-morbidities and Peri-operative Data***				
Priority Status	Emergency	5 (0.2)	6(0.2)	<0.0001
	Elective	2056 (98.5)	457 (88.9)	
	Urgent	26 (1.2)	50 (9.7)	
	Salvage	1 (0.00)	1 (0.2)	
Type of Operation	CABG	2022 (96.8)	506 (98.4)	0.05
	OPCABG	66 (3.2)	8 (1.6)
Pul. Hypertension		8 (0.4)	1 (0.2)	1.0
Angina Class (CCS)*	Class I	278 (13.3)	51 (9.9),	<0.0001
	Class II	175 (8.4)	45 (8.8)	
	Class III	1579 (75.6)	374 (72.8)	
	Class IV	56 (2.7)	44 (8.6)
Pre-op Creatinine levels (mg/dl)		1.00±0.31	1.00±0.24	0.89
Pre-op CKMB Levels (IU/L)		22.91±30.11	23.25±23.37	0.82
Category of Disease	SVD	109 (5.2)	50 (9.7)	<0.0001
	DVD	336 (16.1)	71 (13.8)	
	TVD	1643 (78.70	393 (76.5)
Ejection Fraction (%)		51.02±10.13	52.94±9.72	<0.0001
L.V Function Grades	Grade I	1346 (64.5)	373 (72.6)	0.001
	Grade II	463 (22.2)	97 (18.9)	
	Grade III	279 (13.4)	44 (8.6)
Parsonnet score		4.15±3.58	4.12±4.40	0.86
Add-euro Score		1.18±1.23	1.27±1.37	0.14
Log-euro Score		1.38±.74	1.44±0.89	0.15

* CCS= Canadian Cardiovascular Society.

**Table-II:** Comparison of operative and post-operative characteristics.

Name of Variable		Non-LMS group (Control Group)	LMS Group (Study Group)	P-Value
Bypass time (min.)		108.81±30.47	111.35±32.13	0.114
Cross-Clamp time (min.)		62.83±20.20	64.07±19.90	0.219
Chest Drainage		667.81±381.81	693.84±357.07	0.157
Post-op CKMB* Levels (IU)		61.35±82.43	62.38±64.40	0.785
Duration of Support (hours)		11.08±19.50	14.47±26.25	0.002
Ventilation time (hours)		8.07±23.65	10.52±31.53	0.056
Hospital stay time (days)		7.12±3.16	7.47±3.22	0.032
IABP** (%)		62 (3.0)	29 (5.6)	0.003
Operative Mortality (%)		17 (0.8)	13(2.5)	0.001
Inotropic Support	Mild	1250 (59.9)	264 (51.4)	<0.0001
	Moderate	459 (22.0)	152 (29.6)	
	High Dose	39 (1.9)	28 (5.4)	
	Nil	340 (16.3)	70 (13.6)
Pul. Complications (%)		78 (3.7)	34 (6.6)	0.005
Neurologic complications	Transient Ischemic Attacks	2 (0.1)	2 (0.4)	0.259
	Permanent Local Paralysis	3 (0.1)	1 (0.2)	
	Brain death	3 (0.1)	2 (0.4)	
	Nil	2062 (98.8)	503 (97.9)	
	Localized Paralysis	2 (0.1)	0 (0.0)	
	Acute Confessional State	16 (0.8)	6 (1.2)
Renal Complications (%)		26 (1.2)	5 (1.0)	0.610

* CKMB = Creatinine Kinase Myocardial Band,

** IABP= Intraaortic Balloon Pump.

The need, duration and dose of pharmacological inotropic support and intra-aortic balloon counter-pulsation were significantly higher in Group II (LMS Group). The mean length of ventilation and hospital stay time were significantly higher in group II.

The peak CK-MB levels after surgery in 36 hours were not statistically different in both groups (p=0.785). Operative mortality was also significantly higher in group II (p-value 0.001). There was no difference regarding postoperative neurological and renal complications in both groups.

## DISCUSSION

In the era of medicated stents, stenosis of left main coronary artery still remains unchallenged indication for CABG. Indeed, current American College of Cardiology/American Heart Association (ACC/AHA) guidelines state that for significant LMS stenosis CABG is class I indication even in asymptomatic patients (class A evidence)^5^ and PCI is a class III indication in those who are otherwise eligible for CABG.^9^ In the recent years, the proportion of patient with stenosis of left main coronary artery referred for CABG has therefore increased sharply.

In this study, the mortality of CABG in patients with significant left main coronary stenosis was 2.5% comparable with other reports of an early mortality in the range of 2–5%.^10^,^11^ In our study group about 19.8% patients were with significant LMS disease. In this study, Patients with left main stem disease were older as compared to patients without left main stem disease, because elderly patient population admitted for surgery had more advanced coronary artery disease and more often Left Main Coronary Artery disease than younger patients.^12^,^13^ About 80% patients with significant LMS disease had associated three vessel disease which is one of the limitation of PCI as alternative treatment option.^14^ Several clinical trials^15^-^17^ and data registries^17^,^18^ have revealed comparable procedural risks for both PCI and CABG, but the rates of subsequent re-intervention remained high with PCI in these studies. The complexity of the atherosclerotic lesions also have a prognostic impact on the outcome of PCI as the SYNTAX score has shown, which is not the case for CABG.^19^,^20^

Many studies have showed LMS disease a risk factor for surgery. A review of 7 large data sets, representing more than 172 000 patients who underwent surgery between 1986 and 1994, was carried out to find the predictive power of certain preoperative variables.^21^ Seven core variables (i.e., urgency of operation, age, prior heart surgery, sex, LVEF, percent stenosis of the left main coronary artery, and number of major coronary arteries with more than 70% stenosis) were found to be predictive of mortality after CABG in all 7 data sets. However the different risk estimation scoring system like Additive Euro score and Logistic Euro score, no one has given any additional marks to LMS disease. Our study showed that although the patients in non LMS group had more advanced LV dysfunction and extent of coronary disease but cardiac morbidity and mortality is more in LMS group indicating significant LMS disease as independent operative risk factor for CABG surgery. Although LMS disease had direct and indirect associations with operative morbidity and mortality, the operative results are acceptable and steadily improving.

The higher risk in LMS disease is probably because majority of patients are older and have unstable angina and frequently they need urgent surgery as in our study. The key message in the study is that operative team needs more vigilance in patients with LMS disease during peri-operative period because of above mentioned risk profiles.

### Limitations of the study

Like all retrospective studies, this study has some obvious limitations. It had some inherent limitations of the study design. Angiographic details, such as the anatomical sites of LMS stenosis, were not available. The long-term follow-up data was not studied as the study only encompasses early outcomes. The data revealed increased pulmonary morbidity in patients with LMS disease but the contributing factors have not been studied.

## CONCLUSION

This study showed that significant LMS disease is an independent risk factor for early cardiopulmonary morbidity and mortality after CABG surgery.

## References

[1] Proudfit WL, Shirey EK, Sones FM (1967). Distribution of arterial lesions demonstrated by selective cinecoronary arteriography. Circulation.

[2] Keogh B, Kinsman R (2004). Fifth national adult cardiac surgical database report 2003: improving outcomes for patients: Dendrite Clinical Systems.

[3] Tanabe K, Hoye A, Lemos PA, Aoki J, Arampatzis CA, Saia F (2004). Restenosis rates following bifurcation stenting with sirolimus-eluting stents for de novo narrowings. The Am J Cardiol.

[4] Keogh B, Kinsman R (2003). The Society of Cardiothoracic Surgeons of Great Britain and Ireland. Fifth national adult cardiac surgical database report.

[5] Hillis LD, Smith PK, Anderson JL, Bittl JA, Bridges CR, Byrne JG (2011). 2011 ACCF/AHA guideline for coronary artery bypass graft surgery: A report of the American College of Cardiology Foundation/American Heart Association task force on practice guidelines developed in collaboration with the American Association for Thoracic Surgery, Society of Cardiovascular Anesthesiologists, and Society of Thoracic Surgeons. J Am Coll Cardiol.

[6] Nashef SA, Roques F, Michel P, Gauducheau E, Lemeshow S, Salamon R (1999). European system for cardiac operative risk evaluation (EuroSCORE). Eur J Cardio-Thoracic Surg.

[7] Conley M, Ely R, Kisslo J, Lee K, McNeer J, Rosati R (1978). The prognostic spectrum of left main stenosis. Circulation.

[8] Chaitman BR, Rogers WJ, Davis K, Tyras DH, Berger R, Bourassa MG (1980). Operative risk factors in patients with left main coronary-artery disease. N Eng J Med.

[9] Smith SC, Feldman TE, Hirshfeld JW, Jacobs AK, Kern MJ, King SB (2006). ACC/AHA/SCAI 2005 Guideline Update for Percutaneous Coronary Intervention A Report of the American College of Cardiology/American Heart Association Task Force on Practice Guidelines (ACC/AHA/SCAI Writing Committee to Update the 2001 Guidelines for Percutaneous Coronary Intervention). J Am Coll Cardiol.

[10] Ellis SG, Hill CM, Lytle BW (1998). Spectrum of surgical risk for left main coronary stenoses: benchmark for potentially competing percutaneous therapies. Ame Heart J.

[11] d’Allonnes FR, Corbineau H, Le Breton H, Leclercq C, Leguerrier A, Daubert C (2002). Isolated left main coronary artery stenosis: long term follow up in 106 patients after surgery. Heart.

[12] Disch DL, O’Connor GT, Birkmeyer JD, Olmstead EM, Levy DG, Plume SK (1994). Changes in patients undergoing coronary artery bypass grafting: 1987-1990. Northern New England Cardiovascular Disease Study Group. Ann Thorac Surg.

[13] Edwards FH, Clark RE, Schwartz M (1994). Coronary artery bypass grafting: The Society of Thoracic Surgeons National Database experience. Ann Thorac Surg.

[14] Taggart DP, Kaul S, Boden WE, Ferguson TB, Guyton RA, Mack MJ (2008). Revascularization for unprotected left main stem coronary artery stenosisStenting or surgery. J Am Coll Cardiol.

[15] Park S-J, Kim Y-H, Park D-W, Yun S-C, Ahn J-M, Song HG (2011). Randomized trial of stents versus bypass surgery for left main coronary artery disease. N Eng J Med.

[16] Seung KB, Park D-W, Kim Y-H, Lee S-W, Lee CW, Hong MK (2008). Stents versus coronary-artery bypass grafting for left main coronary artery disease. N Eng J Med.

[17] Park DW, Seung KB, Kim YH, Lee JY, Kim WJ, Kang SJ (2010). Long-term safety and efficacy of stenting versus coronary artery bypass grafting for unprotected left main coronary artery disease: 5-year results from the MAIN-COMPARE (Revascularization for Unprotected Left Main Coronary Artery Stenosis: Comparison of Percutaneous Coronary Angioplasty Versus Surgical Revascularization) registry. J Am Coll Cardiol.

[18] Caggegi A, Capodanno D, Capranzano P, Chisari A, Ministeri M, Mangiameli A (2011). Comparison of one-year outcomes of percutaneous coronary intervention versus coronary artery bypass grafting in patients with unprotected left main coronary artery disease and acute coronary syndromes (from the CUSTOMIZE Registry). Am J Cardiol.

[19] Serruys PW, Morice M-C, Kappetein AP, Colombo A, Holmes DR, Mack MJ (2009). Percutaneous coronary intervention versus coronary-artery bypass grafting for severe coronary artery disease. N Eng J Med.

[20] Park D-W, Kim Y-H, Yun S-C, Song HG, Ahn J-M, Oh J-H (2011). Complexity of atherosclerotic coronary artery disease and long-term outcomes in patients with unprotected left main disease treated with drug-eluting stents or coronary artery bypass grafting. J Am Coll Cardiol.

[21] Jones RH, Hannan EL, Hammermeister KE, DeLong ER, O’Connor GT, Luepker RV (1996). Identification of preoperative variables needed for risk adjustment of short-term mortality after coronary artery bypass graft surgery. J Am Coll Cardiol.

